# Development of an Electrochemical Sensor Based on Molecularly Imprinted Polymer Using Functionalized Gold Nanoparticles for Caffeine Quantification

**DOI:** 10.3390/bios15100704

**Published:** 2025-10-18

**Authors:** Sergio Espinoza-Torres, Astrid Choquehuanca-Azaña, Marcos Rufino, Eleilton da Silva, Lucio Angnes

**Affiliations:** 1 Department of Fundamental Chemistry, Institute of Chemistry, University of São Paulo, Av. Prof. Lineu Prestes, 748, São Paulo 05508-000, SP, Brazil; 2 Faculty of Pharmaceutical Sciences, Faculdades Oswaldo Cruz, Rua Brigadeiro Galvão, 540, São Paulo 01151-000, SP, Brazil

**Keywords:** caffeine, molecularly imprinted polymer, electrochemical sensor, electropolymerization

## Abstract

Caffeine is a natural alkaloid consumed primarily for its stimulant and metabolic effects. Some everyday products, such as coffee, tea, soft drinks, sports supplements, and even pain relievers, contain caffeine. However, excessive caffeine consumption, greater than 400 mg per day, can cause adverse effects. Therefore, this work presents an electrochemical sensor based on a molecularly imprinted polymer (MIP) electropolymerized on gold nanoparticles functionalized with p-aminothiophenol (AuNPs-pATP) for caffeine quantification. AuNPs-pATP synthesized show a spherical morphology with an average diameter of 2.54 nm. Stages of MIP formation were monitored by cyclic voltammetry (CV) and electrochemical impedance spectroscopy (EIS) using a potassium ferrocyanide redox probe, where the following were observed: (i) an increase in conductivity upon modification of the GCE with AuNPs-pATP, (ii) the blocking of active sites during the electropolymerization step, and (iii) the release of specific cavities upon template removal, revealing consistent differences between the MIP and the control polymer (NIP). SEM images revealed three-dimensional spherical cavities on MIP surface, while the NIP showed a more compact rough surface. Caffeine quantification was performed using square wave voltammetry (SWV) with LOD of 0.195 µmol L^−1^ and LOQ of 0.592 µmol L^−1^. Interference studies indicated high selectivity and a high density of caffeine-specific binding sites in the MIP. Additionally, MIP sensor demonstrated reusability, good reproducibility, and stability, as well as promising results for analysis in soft drink and sports supplement samples.

## 1. Introduction

Caffeine (1,3,7-trimethylxanthine) is a member of the methylxanthine class of alkaloids that includes theobromine and theophylline. It is a natural constituent of more than 60 plant species, including coffee beans, tea leaves, cola nuts, cacao, yerba mate, and guarana berries [1]. Caffeine (CAF) is one of the most consumed food ingredients worldwide, found in beverages such as coffee, tea, energy drinks, and carbonated drinks, as well as in products containing cocoa or chocolate [2,3]. It is also found in a variety of medications and dietary supplements, as it has psychoactive properties, allowing its use as a central nervous system (CNS) stimulant, diuretic, and in analgesic compounds [4,5]. So, caffeine has several positive effects, including reduced fatigue, pain relief, decreased drowsiness, increased alertness, and concentration, suppressed appetite, slightly reduced weight gain, and decreased risk of depression and suicide [6,7,8]. However, excessive consumption can cause anxiety, agitation, gastrointestinal disorders, high blood pressure, cardiovascular disease, disrupt sleep patterns, tremors, nausea, hyperactivity, seizures, kidney dysfunction, induce daily headaches, impair normal child development, and have adverse effects on fertility [3]. In pregnancy, the main concerns are induction of spontaneous abortion and poor fetal growth [6,9]. Furthermore, it is important to understand that caffeine is a mild CNS stimulant, but when combined with other stimulants, it increases the risk of agitation, tremor, insomnia, and seizures [4]. The U.S. Food and Drug Administration (FDA) recommend ≤ 400 mg of caffeine per day for a healthy adult [10,11]. It has been reported that the presence of caffeine in aquatic ecosystems has affected the development and reproduction of species, even causing lethality [12]. Therefore, the importance of detecting and quantifying caffeine encourages the ongoing search for new analytical methods capable of detecting this compound accurately and quickly [13,14].

Although electrochemical methods offer several advantages (portability, high sensitivity, short analysis time, low cost, etc.), chromatographic and spectroscopic methods are the most widely used for caffeine quantification [6,7,15,16,17]. The main disadvantage of electrochemical methods is selectivity, as certain components of a studied matrix can significantly interfere with the desired detection. To solve selectivity problems, recognition materials, such as molecularly imprinted polymers (MIPs), aptamers, enzymes, and antibodies, have been introduced into electrode modifications [10,18].

The unique properties of MIPs, such as low cost, ease of preparation, controllable morphology, high chemical and physical stability, sensitivity in complex matrices and high selectivity, have sparked increased interest among scientists for molecular recognition applications [11,13]. However, MIPs polymerized by traditional methods (bulk, precipitation, sol–gel, etc.) have limitations, such as incomplete template removal, difficult accessibility to the binding site, slow interaction kinetics, and the formation of heterogeneous cavities. Therefore, the preparation of MIPs by electropolymerization is positioned as the most efficient method because it is simple, rapid, reproducible, and environmentally friendly [2].

There are some works based on molecular imprinting technology for caffeine quantification; among them, one uses polypyrrole synthesized by electropolymerization, in the presence of caffeine, as a template, on the surface of a glassy carbon electrode (GCE) [19]. Another study presents a novel electrochemical sensor that was fabricated on the surface of a pencil graphite electrode (PGE) by one-step electropolymerization with gold nanoparticles (AuNPs) and caffeine. This combination, like the MIP thin film with AuNPs, improves the electrical response by facilitating charge transfer processes [2]. A third approach presents the integration of a MIP into a carbon paste electrode, which functioned as a selective recognition element and preconcentrating agent for the detection of CAF [20].

In the present study, we constructed an analytical device for caffeine detection based on MIP associated with AuNPs-pATP. The incorporation of AuNPs functionalized with pATP into MIP-based electrochemical sensors allows us to efficiently combine the advantages of both materials. The main advantages of using this MIP sensor compared to other sensors are (i) the ease and speed of preparing the MIP by electropolymerization, since in traditional methods (bulk, sol–gel, precipitation, etc.), the removal of the template can take many days, generating abundant amounts of toxic waste due to the excessive use of organic solvents, (ii) the layer-by-layer modification of the electrode using functionalized gold nanoparticles that guarantees greater homogeneity of the binding sites, in addition to the same orientation and location on the surface due to the amine groups of the functionalization [21], and (iii) indirect quantification using a known redox probe at potentials close to 0 V to avoid reaching high positive potentials (1.5 V) where caffeine oxidation occurs [19,20].

**Figure 1:**
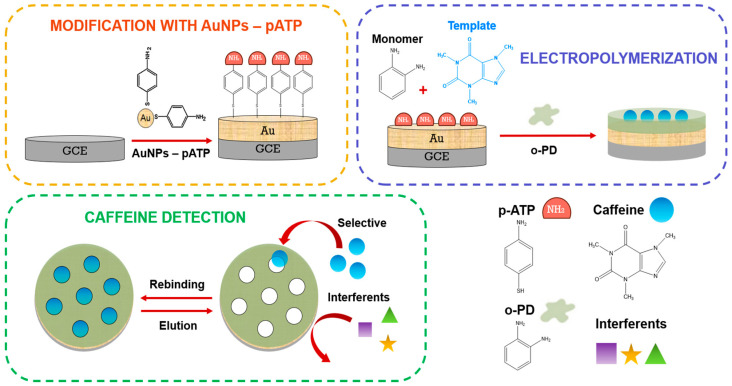
Schematic illustration of the preparation of electropolymerized MIP film and its application for the quantification of caffeine.

The synthesis of the gold nanoparticles functionalized with p-aminothiophenol was evidenced by UV-vis and IR characterizations. The construction of the electrochemical sensor (Figure 1) began with the modification of the GCE with the functionalized gold NPs by drop casting. Then, on the modified electrode, electropolymerization was performed using caffeine as template and o-phenylenediamine as monomer. To monitor each step during the formation of MIP and caffeine presence in MIP cavities, [Fe(CN)_6_]^3−/4−^ was used as a redox probe, where the current response decreases as the cavities are occupied. Additionally, parameters such as polymerization cycles, elution time, and rebinding time were optimized, and interferents, reuse, reproducibility, and stability studies were performed. Finally, the detection of caffeine in real samples was evaluated.

## 2. Materials and Methods

### 2.1. Chemicals

Caffeine (CAF), p-aminothiophenol (pATP), o-phelynedediamine (oPD), tetrachloroauric acid (HAuCl_4_), sodium borohydride (NaBH_4_), methanol, glacial acetic acid, potassium ferrocyanide (K_4_[Fe(CN)_6_]), potassium ferricyanide (K_3_[Fe(CN)_6_], and for selectivity tests theobromine, xanthine, and glucose were used, were purchased from Sigma-Aldrich.

Phosphate-buffered saline (0.1 mol L^−1^, pH 7.0) was prepared using disodium phosphate and sodium hydrogen phosphate. All aqueous solutions were prepared using water purified with a Milli-Q purification system (resistivity ≥ 18 MΩ cm).

Electrochemical measurements were performed using a Autolab potentiostat–galvanostat (Hach Lange, France), monitored by the NOVA 2.1 software (Metrohm, Netherland). All electrochemical experiments were performed in a three-electrode cell containing 5 mL of electrolyte solution, with a glassy carbon working electrode (0.07 cm^2^), an Ag/AgCl-saturated reference electrode, and a platinum plate as the auxiliary electrode. All experiments were carried out at room temperature (25 °C).

Infrared spectra and absorbance spectra were obtained using a Bruker ALPHA Fourier Transform Infrared Spectroscopy in Attenuated Total Reflectance (ATR) mode (Billerica, MA, USA) and UV-vis spectrophotometer (SEIKO-SPA400, Seiko instruments Inc., Chiba, Japan), respectively. The JEM 2100 JEOL transmission electron microscope with a LaB6 filament was used to register the TEM images of pATP-functionalized AuNPs. Scanning electron microscope (SEM) measurements were performed on a 5–15 kV Thermo Fisher Scientific Inspect F50 (Waltham, MA, USA). The SEM measurements for GCE/AuNPs-pATP/MIP and GCE/AuNPs-pATP/NIP were performed using an accelerating voltage of usually 5 kV with a working distance of ~8 mm.

### 2.2. Synthesis of pATP-Functionalized AuNPs

The gold nanoparticles functionalized were synthesized by chemical reduction using a sodium borohydride solution as reductant agent. For this purpose, 1.6 × 10^−4^ mol of tetrachloroauric (III) acid trihydrate was dissolved in 60 mL of methanol. Then, a solution of pATP was prepared dissolving 1.6 × 10^−4^ mol in 12 mL of methanol and water in a ratio of 1:1 (*v*/*v*) and added dropwise under stirring to the gold salt solution. After 10 min, a solution of 8.0 × 10^−4^ mol of NaBH_4_ in 2.2 mL of water was added dropwise to the mix under vigorous stirring. Then, the solution was kept in darkness without stirring for 1 h. The resulting suspension was filtered under vacuum and washed successively with water and ethanol. Finally, the powder was dried and stored as a solid [22].

### 2.3. Caffeine–MIP Electrochemical Sensor Fabrication

Prior to surface modification, the bare GCE was polished with 1.0, 0.3, and 0.05 µm alumina–water slurry and ultrasonicated in HNO_3_, ethanol, and distilled water for 10 min. Before the synthesis of MIP, the working electrode was modified by drop casting of 1.0 µL of AuNPs-pATP (5 mg mL^−1^ in distilled water). Then, the GCE modified (GCE/AuNPs-pATP) was carried out by electropolymerization of monomer oPD (6.0 mmol L^−1^) in a deoxygenated solution in the presence of caffeine (1.0 mmol L^−1^) as a template, using a cyclic voltammetry technique over a potential range of 0.0 to 1.0 V at a scan rate of 50 mV s^−1^ for 10 cycles [23]. This sensor is now called GCE/AuNPs-pATP/PPD.

The next step was to remove the template (CAF) from the imprinted polymer by extraction using a mixture of 0.25 mol L^−1^ NaOH in ethanol/water 1:1 (*v*/*v*) for 15 minutes by magnetic stirring [24]. The obtained sensor was named GCE/AuNPs-pATP/MIP. For the other hand, as a control polymer, a non-imprinted polymer was made in a similar way without addition of the CAF template; it was named GCE/AuNPs-pATP/NIP.

### 2.4. Quantification of Caffeine Using the Caffeine–MIP Electrochemical Sensor

The quantification of caffeine was carried out using square wave voltammetry (SWV) with a modulation amplitude of 20 mV, step of 1 mV, and a frequency of 10 Hz. The caffeine–MIP electrochemical sensor was immersed for 20 min in a solution of caffeine in 0.1 mol L^−1^ PBS 7.0. A potential of 0 to 0.5 V was applied to quantify the concentration of caffeine by the SWV cathodic peak current. For analysis in real samples, dilutions of 1.0 μmol L^−1^ of CAF in Coca-Cola, Red Bull, and capsules in 0.1 mol L^−1^ PBS 7.0 were used. All experiments were performed in triplicate.

### 2.5. Quantification of Caffeine Using HPLC

The caffeine quantification by reverse-phase chromatography was performed using a Shimadzu LC-10 system. A C18 Eclipse XDB column (150 × 4.6 mm, 5 µm) from Agilent was used as the stationary phase. The mobile phase consisted of ultra-pure water (Milli-Q) acidified with acetic acid (99%, Sigma-Aldrich, St. Louis, MO, USA) to pH 4.0 (solvent A) and methanol (99.9%, Carlo Erba, Le Vaudreuil, France) as the organic phase (solvent B). The method used was isocratic, with a flow rate of 1.5 mL min^−1^, using 30% solvent B, at a temperature of 40 °C, and an injection volume of 20 µL [25].

## 3. Results and Discussions

### 3.1. Characterization Experiments

**Figure 2:**
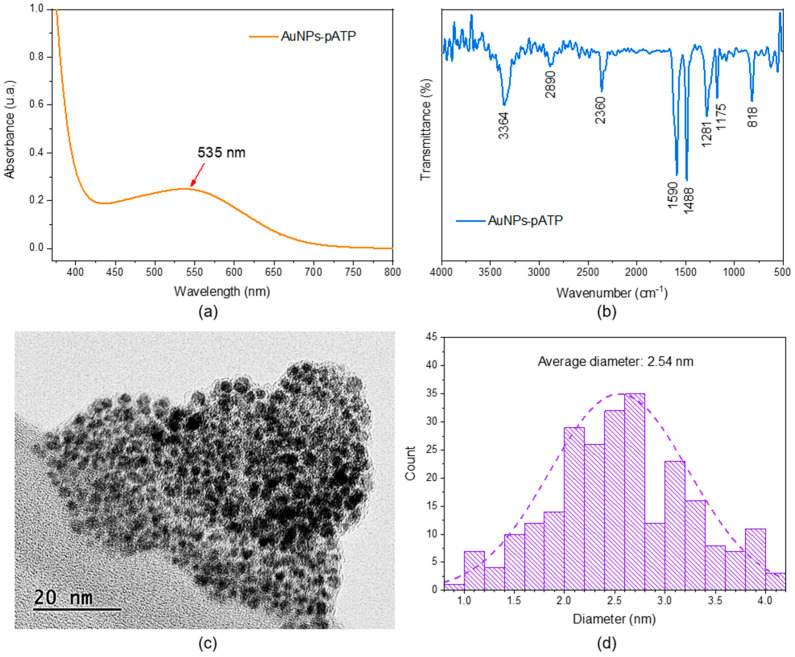
(**a**) UV-vis spectra, (**b**) FT-IR spectra, (**c**) TEM imagen, and (**d**) size distribution of AuNPs-pATP.

The UV-vis absorption spectrum of the AuNPs-pATP presented in Figure 2a shows a broad absorption band at 535 nm, characteristic of this type of functionalization. This result demonstrates that the thiol group (-SH) of the pATP molecules effectively bound to the surfaces of the Au nanoparticles, causing a broadening and shifting of the band toward longer wavelengths compared to pure AuNPs, which usually present a well-defined LSPR band at 520 nm [26,27,28]. Additionally, the shift in the maximum wavelength of the AuNPs to a longer wavelength region indicates a possible change in the morphology and a certain degree of aggregation after their functionalization [29].

The FTIR spectrum of AuNPs-pATP (Figure 2b) shows characteristic peaks of pATP in 3364 cm^−1^ corresponding to the stretching vibration of –N–H of the charged amine. The aromatic –C=C– in-plane vibrations appeared at 1593 and 1491 cm^−1^, and these peaks confirmed the presence of a benzene ring on AuNPs surfaces. The characteristic band at 818 cm^-1^ was assigned to the vibration of the =C-H of the benzene ring [29].

The TEM images presented (Figure 2c) show the formation of very small spherical nanoparticles with an average diameter of 2.54 nm, according to the size distribution obtained using ImageJ 1.8.0 software (Figure 2d), this result is consistent with some previously reported work [22,30,31]. However, it is important to mention that due to the high affinity of pATP for gold, pATP molecules are expected to bind not only to the open surface of the nanoparticles, but also to sites located between neighboring nanoparticles [26]. Therefore, Figure S1 shows that the functionalization wraps around the AuNPs, forming small elliptical agglomerates with a major diameter between 20 and 50 nm, this being supported by the UV-vis adsorption spectrum obtained (Figure 2a).

### 3.2. Caffeine Sensor Modification

For the fabrication of a high-quality poly-o-phenylenediamine polymeric film, proper adhesion of the monomer to the substrate is essential. AuNPs-pATP was used to modify the electrode surface, since it contributes to improving the electrical properties of the sensor, while facilitating strong adsorption and increasing the reaction activity of the surface due to the strong interaction between gold and the thiol group (-SH) of pATP, leaving the amino group (-NH_2_) exposed on the surface capable of immobilizing caffeine through non-covalent interactions [32,33]. Experiments were performed to evaluate the optimal amount of AuNPs-pATP (1 mg mL^−1^) to be deposited on the electrode surface before oPD electropolymerization.

**Figure 3:**
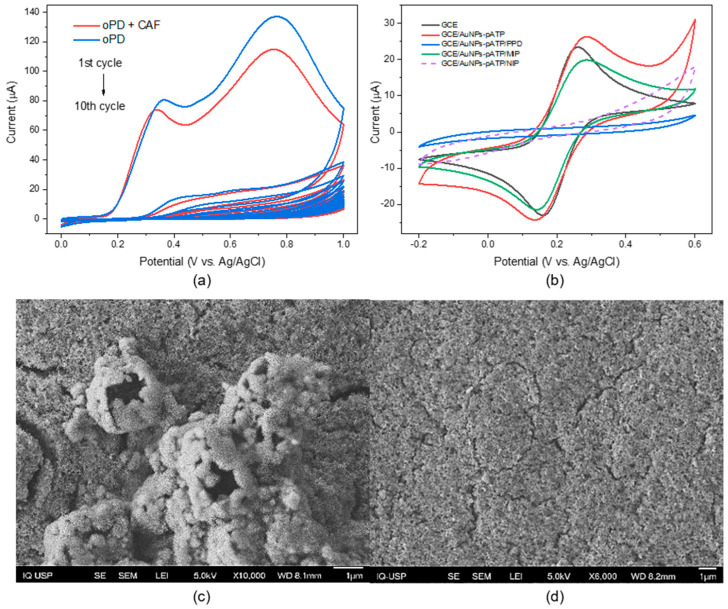
(**a**) Cyclic voltammograms for the polymerization of oPD in 0.1 mol L^−1^ PBS (pH = 7.0) for 10 consecutive cycles with (red) and without CAF (blue) templates at a scan rate of 50 mV s^−1^ and (**b**) CVs of steps of MIP preparation in PBS 7.0 electrolyte containing 5 mmol L^−1^ of [Fe(CN)_6_]^3−/4−^ as a redox probe and 50 mmol L^−1^ of KCl. SEM imagens of (**c**) GCE/AuNPs-pATP/MIP and (**d**) GCE/AuNPs-pATP/NIP.

Figure 3a shows the CV recorded during the electropolymerization of oPD in the presence and absence of CAF. In the first cycle, we can see two oxidation peaks, at 0.35 V and 0.75 V. The current peak at 0.35 V corresponds to the oxidation of the oPD monomer, which gradually decreases with the increasing number of cycles and confirms the formation of a PPD film on the electrode surface. Furthermore, the current peaks with template (CAF) are lower than those without CAF, due to the introduction of CAF into the PPD film during electropolymerization [24].

Figure 3b shows cyclic voltammograms of each electrode modification step. The presence of functionalized gold nanoparticles increases the conductivity of the sensing layer, as well as the number of binding sites through enhanced roughness of the surface [34]. Electrode modification using functionalized gold nanoparticles results in an increase in the redox peak pair compared to the unmodified electrode. Once the polymerization is complete, the oxidation peaks disappear, demonstrating the formation of the poorly conductive PPD film [24]. After removing the caffeine from the PPD film, there is an increase in the peak signal; the NIP, on the other hand, does not suffer a significant increase in the current peaks, showing that the PPD film does not suffer any degradation effects from the washing solution [35]. Impedance spectroscopy was used to characterize the stages of electrode modification (Figure S2). The modification of GCE with AuNPs-pATP (in red) generates an improvement in the charge transfer that decreases after electropolymerization due to the insulating PPD film formed on the surface (in blue). After washing the polymer, the high charge transfer resistance decreases (in green) because cavities are generated in the film due to the caffeine extraction [36].

Additionally, the SEM images obtained for the GCE/AuNPs-pATP/MIP show a rough surface with the formation of well-defined hollow spheres, which can be associated with the formation of the MIP with cavities specific to caffeine (Figure 3c). Meanwhile, for the GCE/AuNPs-pATP/NIP, the surface obtained is more compact and less rough, with no evidence of defined cavities (Figure 3d).

### 3.3. Optimization of Parameters

Before the quantification of CAF with the electrochemical sensor, several parameters including (a) electropolymerization cycles, (b) elution time of template, and (c) rebinding time were optimized by using the peak currents (Ip) by square wave voltammograms.

**Figure 4:**
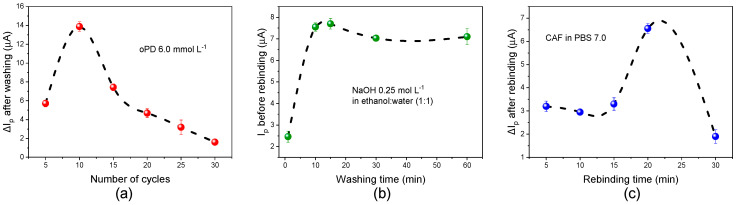
Optimization of parameters of electrochemical sensor: (**a**) electropolymerization cycles, (**b**) washing time, and (**c**) rebinding time, by square wave voltammetry in 0.1 mol L^−1^ PBS 7.0 electrolyte containing 5.0 mmol L^−1^ of [Fe(CN)_6_]^3−/4−^ as a redox probe.

Figure 4a shows the relationship between electropolymerization cycles and peak current before rebinding, scan cycles were used to evaluate the MIP film thickness. We can observe a low current in less than 10 cycles; under these conditions, the film created is very thin, as well as not allowing creation of sufficient imprinted cavities. However, a higher number of cycles creates a thicker film with a less conductive surface and makes it difficult to remove the template from the center of the polymer. Therefore, 10 polymerization cycles were the optimal condition found, where the current also reached its maximum value. The washing time was also optimized (Figure 4b), with an increase in current observed up to 15 minutes, after which small decreases are observed, which may be due to wear of the PPD film due to excessive washing. Rebinding time was also optimized at 20 minutes as the time to fill the largest number of selective cavities (Figure 4c); longer rebinding times result in a decrease in current due to polymer swelling, which can distort or resize the printed cavities and weaken existing non-covalent interactions [37].

### 3.4. Detection of Caffeine (CAF)

The electrochemical sensor was used for the quantification of CAF via indirect detection. Square wave voltammograms of GCE/AuNPs-ATP/MIP were recorded before and after the rebinding of CAF, using 5.0 mmol L^−1^ of [Fe(CN)_6_]^3−/4−^, a redox probe to evaluate the filling of imprinted cavities. Indirect detection was chosen because direct electrochemical determination of caffeine using common electrode materials such as metals or glassy carbon occurs at a very positive potential (around 1.5 V), which overlaps with the discharge of the background medium [20].

**Figure 5:**
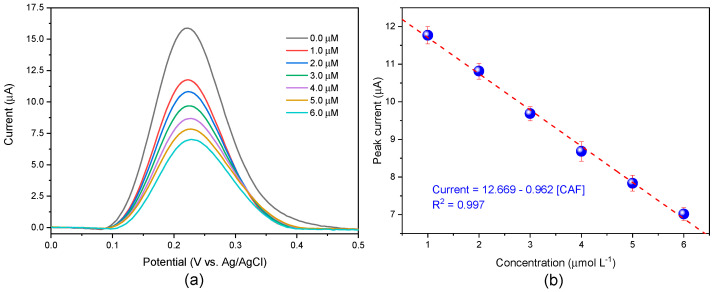
(**a**) Square wave voltammograms of GCE/AuNPs-ATP/MIP in 0.1 mol L^−1^ PBS 7.0 electrolyte containing 5.0 mmol L^−1^ of [Fe(CN)_6_]^3−/4−^ as a redox probe after the rebinding of CAF at different concentrations for 20 min. (**b**) Linear relationship between peak currents and CAF concentrations.

The Ip decreases gradually with increasing concentration of CAF, this occurs because the selective cavities formed in the MIP are again occupied by CAF, which leads to greater resistance to charge transfer on the electrode surface (Figure 5a).

As shown in Figure 5b, the value of Ip is correlated linearly with the concentration of CAF from 1.0 to 6.0 μmol L^−1^. The linear equation can be expressed as Ip (μA) = (12.669 ± 0.106)–(0.962 ± 0.027) CAF (μmol L^−1^), with coefficient of determination of R^2^ = 0.997, and the limit of detection (LOD) of 0.195 μmol L^−1^, and limit of quantification (LOQ) of 0.592 μmol L^−1^.

### 3.5. Selectivity, Reuse, Reproducibility, and Stability

The selectivity of the electrochemical sensor was evaluated by comparing the decrease in peak current (ΔIp) against CAF and some interfering species such as xanthine and theobromine (similar in structure to CAF) (Figure S3) and glucose, citric acid, and NaCl (common compounds in beverages with CAF).

**Figure 6:**
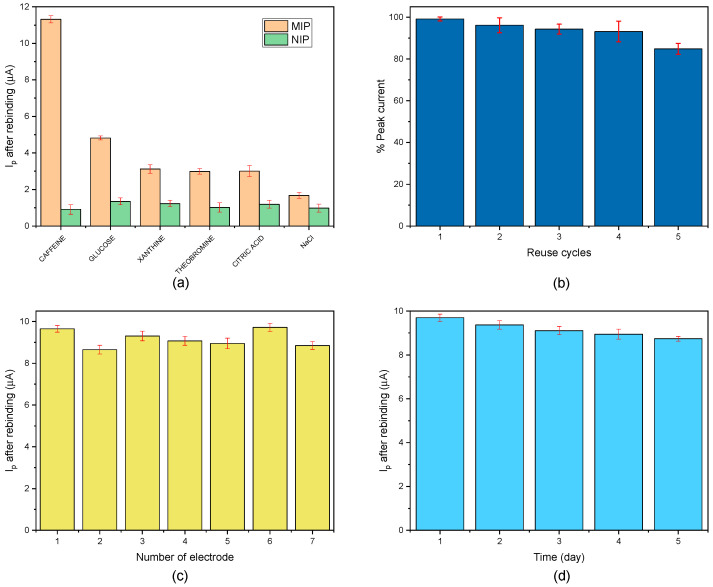
(**a**) Selectivity, (**b**) reuse, (**c**) reproducibility, and (**d**) stability of the imprinted electrochemical sensor.

Figure 6a shows how the current decrease (ΔIp) against these interferents is significantly lower than that against CAF, indicating the high selectivity sites of the electrochemical sensor. The relationship between the MIP and NIP currents provides us with the imprinting factors (IF), where the value for caffeine is significantly higher than that obtained for the interferents, highlighting the correct formation of the printed cavities and their excellent selectivity. Furthermore, the selectivity factor (β) greater than 1 suggests better orientation of functional groups in the formation of the selective imprinted cavities to caffeine (Table 1) [38]. On the other hand, Figure 6b shows the current percentage values for five reuse cycles of the sensor after rebinding to the CAF in the imprinted cavities and subsequent washing in NaOH 0.25 mol L^−1^ in ethanol/water (1:1) for 15 minutes for reuse. The Ip percentage values vary with a small relative standard deviation (RSD) of 3.61%, demonstrating excellent reuse capacity of the imprinted CAF sensor. Figure 6c shows the Ip values of seven imprinted sensors that were prepared under the same conditions and tested with the rebinding of CAF. The relative standard deviation (RSD) of 4.42% demonstrates an imprinted sensor with good reproducibility. Finally, the stability tests (Figure 6d) show a decrease to 90.10% of the initial peak current value after five days, demonstrating good stability of the sensor.

### 3.6. Detection of CAF in Real Samples

Finally, the imprinted electrochemical sensor was used for the detection of CAF in the soft drink (Coca-Cola), energy drink (Red Bull), and sports supplement (capsules) samples. For this purpose, caffeine concentrations of 1.0 μmol L^−1^ were used, along with optimized parameters for quantification.

HPLC was used in order to compare the results obtained by electrochemical detection. A diode array detector (DAD) was used, with the wavelength set to 275 nm. Figure S4 shows the chromatographic profiles of each real sample, highlighting the caffeine peak obtained at a 3-minute retention time. The caffeine capsule does not show the presence of other species, unlike Red Bull and Coca-Cola, where the presence of other polar molecules is observed, although outside the measurement range of caffeine. A calibration curve with 10 points was constructed, ranging from 1 to 100 mg L^−1^ (Figure S5). The limit of detection (LOD) and limit of quantification (LOQ) were determined to be 0.82 μmol L^−1^ and 2.67 μmol L^−1^, respectively. Samples were prepared by dilution: Coca-Cola and the energy drink were diluted 10 times, while the internal paste of the tablet was diluted 20 times. At the end, the samples showed the following caffeine concentrations: Coca-Cola (93.0 ± 0.5) mg L^−1^, energetic drink (306 ± 2) mg L^−1^, and caffeine capsule (163.62 mg ± 1.2) mg g^−1^.

The sensor recovery results compared with the HPLC method showed lower recovery percentages for the Coca-Cola sample because the presence of phosphoric acid in its composition possibly generates greater acidity that can interfere with the quantification of caffeine. The overall results are shown in Table 2 and demonstrate the high accuracy of the developed imprinted CAF sensor.

## 4. Conclusions

This work reported the development and application of a MIP-based electrochemical sensor (GCE/AuNPs-pATP/MIP) for the quantification of caffeine in beverage and sports supplement samples. Gold nanoparticles functionalized with pATP were successfully synthesized, as evidenced by FTIR spectroscopy, UV-visible spectrophotometry, and TEM. Additionally, MIP and NIP sensors were characterized by SEM. Caffeine quantification parameters were optimized to 10 polymerization cycles, 15 minutes of elution time, and 20 minutes of rebinding time. In these optimized conditions, the linear range obtained was from 1.0 to 6.0 μmol L^−1^, LOD and LOQ were 0.195 μmol L^−1^ and 0.592 μmol L^−1^, respectively. The sensor showed good selectivity (IF = 12.43) against interferents with structures similar to caffeine and other common compounds found in real samples. Furthermore, its stability, reproducibility (RSD = 4.42%), and reuse (RSD = 3.61%) in five consecutive reuse cycles were verified.

There are few studies based on MIPs for the detection of caffeine in the literature, which makes this analyte even more interesting. Table S1 presents an MIP-modified carbon paste electrode that employs bulk polymerization and template removal using methanol extraction for 48 h generating large amounts of organic waste. A sensor based on polypyrrole NPs is also shown where caffeine detection is achieved at a high oxidation potential using a direct method. Additionally, a MIP/PGE nanocomposite is prepared by a sol–gel method and electropolymerization in a single step without employing a caffeine immobilization step, so the formation of homogeneous and oriented binding sites on the surface is not guaranteed. In contrast to the disadvantages mentioned in previous work, our proposed sensor stands out for its rapid and environmentally friendly synthesis, low cost, use of low detection potentials, the incorporation of AuNPs-pATP for caffeine immobilization, and the efficient formation of selective cavities.

To fully exploit the potential of this type of sensor, future research should focus on exploring different functionalization and new platforms such as polyimide, paper-based electrodes, and polylactic acid (PLA) (3D printing) for portable and scalable selective sensing.

## Figures and Tables

**Table 1:** Results obtained from the analysis of selectivity of MIP and NIP to caffeine in the presence of interfering molecules.

Molecule	ΔI_MIP_ (µA)	ΔI_NIP_ (µA)	IF ^(1)^	β ^(2)^
Caffeine	11.31	0.91	12.43	1.00
Glucose	4.82	1.35	3.57	3.48
Xanthine	3.12	1.23	2.54	4.90
Theobromine	2.99	1.02	2.93	4.24
Citric acid	3.01	1.19	2.53	4.91
NaCl	1.68	0.98	1.71	7.25

^(1)^ IF = ΔI_MIP_/ΔI_NIP_, ^(2)^ β = IF_CAF_/IF_INT_

**Table 2:** Comparison of caffeine detection by HPLC and electrochemical sensor.

Sample	Caffeine Expected	Caffeine Measured by HPLC	Caffeine Measured by Sensor	% Recovery of Sensor (HPLC *)	
Coca-Cola	<150 mg L^−1 (1)^	93.04 mg L^−1^	72.57 mg L^−1^	-	(77.99 *)
Red Bull	320 mg L^−1^	305.99 mg L^−1^	298.24 mg L^−1^	93.2	(97.47 *)
Caffeine capsules	200 mg	163.62 mg	142.40 mg	71.2	(87.03 *)

^(1)^ There is no exact value of caffeine quantity provided by the official Coca-Cola website. * % Recovery of Sensor compared to HPLC measurement.

## Data Availability

The original contributions presented in this study are included in the article/Supplementary Material. Further inquiries can be directed to the corresponding authors.

## Supplementary Materials

The following supporting information can be downloaded at: https://www.mdpi.com/article/10.3390/bios15100704/s1. Figure S1: (a) TEM image of AuNPs-pATP, and size distribution of (b) minor diameter and (c) major diameter of elliptical aggregates. Figure S2: EIS of steps of MIP preparation in PBS 7.0 electrolyte containing 5.0 mmol L^−1^ of [Fe(CN)_6_]^3−/4−^ as a redox probe and 50 mmol L^−1^ of KCl. Figure S3: Structures of (a) caffeine, (b) xanthine, and (c) theobromine. Figure S4: Chromatographic profile of caffeine in (a) Coca-Cola, (b) Red Bull, and (c) Caffeine capsule. Figure S5: Calibration curve by HPLC in concentrations from 1 to 100 mg L^−1^ at 275 nm using diode array detector (DAD). Table S1: Comparison of different electrochemical sensor MIP based for determination of caffeine.

## Abbreviations

The following abbreviations are used in this manuscript:

CAF	Caffeine
MIP	Molecularly Imprinted Polymer
NIP	Non Imprinted Polymer
AuNPs	Gold nanoparticles
oPD	o-Phenylendiamine
pATP	p-Aminothiophenol
CV	Cyclic Voltammetry
SWV	Square Wave Voltammetry
LOD	Limit of Detection
LOQ	Limit of Quantification
