# Supplementary material for: Development of an Electrochemical Sensor Based on Molecularly Imprinted Polymer Using Functionalized Gold Nanoparticles for Caffeine Quantification
Source: Biosensors (Basel). 2025 Oct 18;15(10):704. doi: 10.3390/bios15100704 (PMC12563842; doi:10.3390/bios15100704)
Supplement: Supplementary file 1 [file biosensors-15-00704-s001.zip › biosensors-3881071-supplementary.pdf]

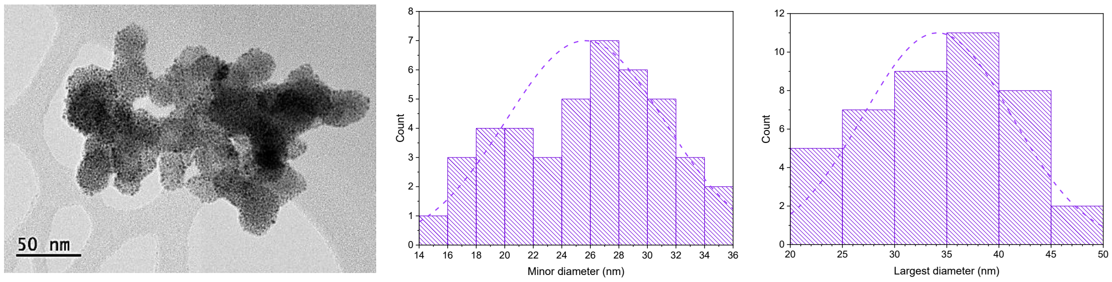

(a) (b) (c)

Figure S1. (a) TEM image of AuNPs-pATP, and size distribution of (b) minor diameter and (c) largest diameter of elliptical aggregates

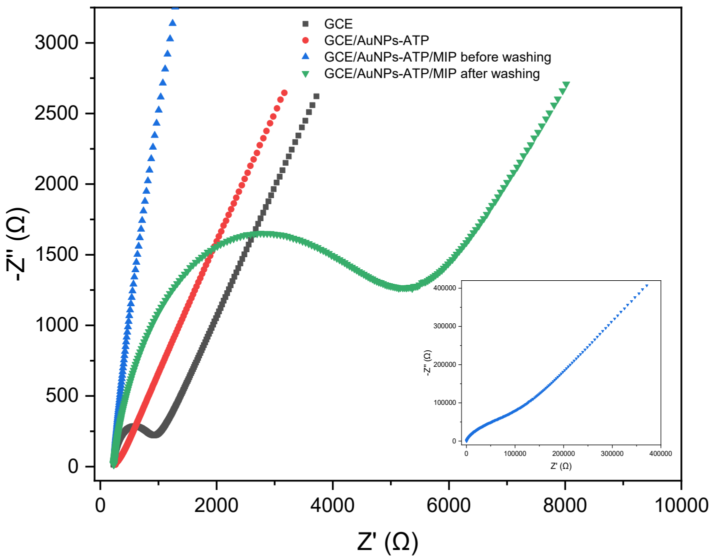

Figure S2. EIS of steps of MIP preparation in PBS 7.0 electrolyte containing 5.0 mmol L^{-1} of $[Fe(CN)_6]^{3-/4-}$ as a redox probe and 50 mmol L^{-1} of KCl

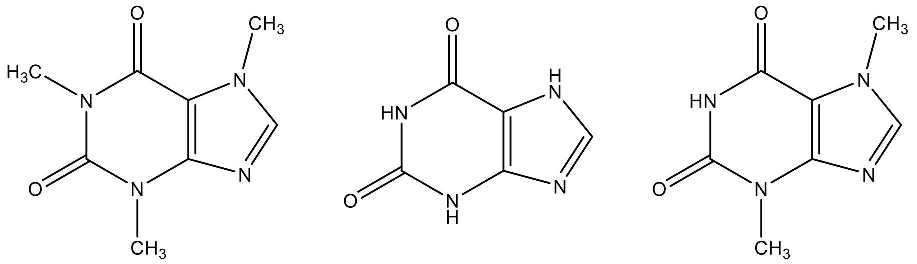

(a) (b) (c)

Figure S3. Structures of (a) caffeine, (b) xanthine and (c) theobromine

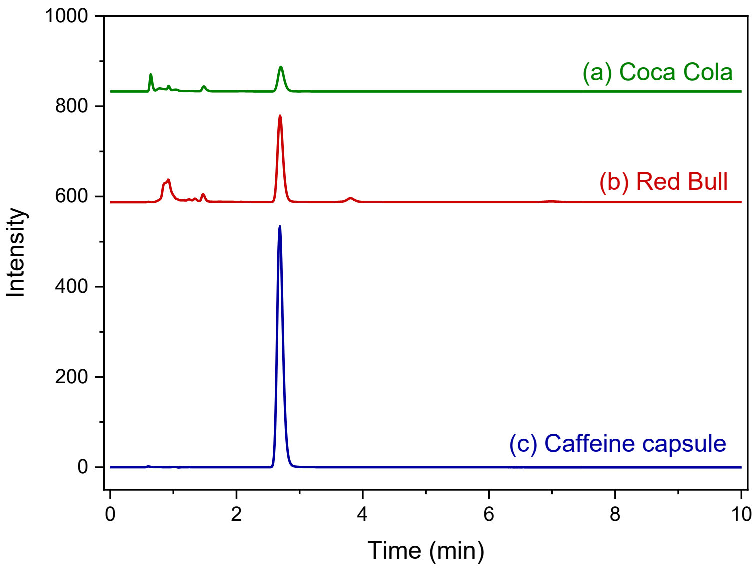

Figure S4. Chromatographic profile of caffeine in (a) Coca Cola, (b) Red Bull and (c) Caffeine capsule

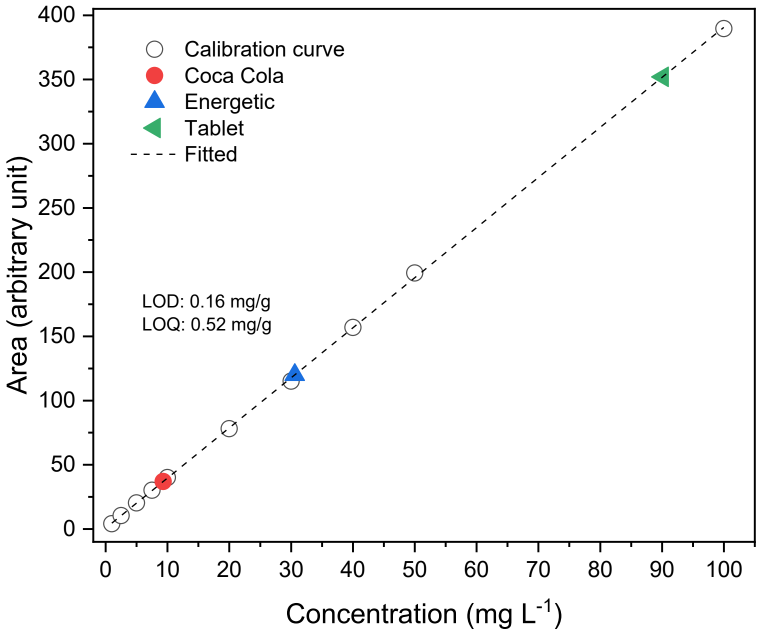

Figure S5. Calibration curve by HPLC in concentrations from 1 to 100 mg L^{-1} at 275 nm using diode array detector (DAD)

Table S1. Comparison of different electrochemical sensor MIP based for determination of caffeine

**Electrode (detection technique)**	**Synthesis method**	**Template removal**	**Performance parameters (LR, LOD, LOQ and IF)**	**Stability and reuse cycles**	**Ref.**
MIP-CP (DPV)	Bulk polymerization	Soxhlet extraction with methanol (48 h)	LR: 6×10^{-8} to 2.5×10^{-5} mol L^{-1}<br>LOD: 1.5×10^{-8} mol L^{-1}	5 weeks and five repeated	[20]
GCE/PPy NPs (DPV)	Electro-polymerization	Ethanol/acetic acid (9:1 v/v) solution	LR: 30 to 150 µmol L^{-1}<br>LOD: 2.5 µmol L^{-1}	10 reuse cycles	[19]
Nanocomposite MIP/PGE (SWV)	Electro-polymerization	Acetic acid (50% V/V)	LR: 0.002 to 0.05 µmol L^{-1} and 0.05 to 1 µmol L^{-1}<br>LOD: 0.9 nmol L^{-1}<br>IF: 12.7	14 days and 12 reuse cycles	[2]
GCE/AuNPs-pATP/MIP (SWV)	Electro-polymerization	NaOH 0.25 mol L^{-1} in ethanol:water (1:1)	LR: 1.0 to 6.0 µmol L^{-1}<br>LOD: 0.195 µmol L^{-1}<br>LOQ: 0.592 µmol L^{-1}<br>IF: 12.43	5 days of stability and 5 reuse cycles	This work
